# 

**Published:** 1893-07-22

**Authors:** 


					trn&Um *> w Ditto po* Annuo. 8o., post fMo
?t?rimna in the United Kingdom and Abroad.]
WffUyxIftA NltrtaiHH tiUPPLEUlkHI,
(No bice twopence. rr- W t-
Vol. XIV.?July 22nd, 1893. V MTm, .M& t Monthixp*rto.??.i ?>.t&oo.
l&tol.t?, ....  J_ J  . lit, c? ?> dgkiBg> W Ditto not ium 8a-. post free
V
356! Vol. XIV. Saturday,
Jttly 22nd, 1893.
[Twopence.

				

